# Quantum dot cascade laser

**DOI:** 10.1186/1556-276X-9-144

**Published:** 2014-03-25

**Authors:** Ning Zhuo, Feng Qi Liu, Jin Chuan Zhang, Li Jun Wang, Jun Qi Liu, Shen Qiang Zhai, Zhan Guo Wang

**Affiliations:** 1 Key Laboratory of Semiconductor Materials Science, Institute of Semiconductors, Chinese Academy of Sciences, P. O. Box 912, Beijing 100083, China

**Keywords:** Quantum dot, Quantum cascade laser, MBE, Mid-infrared

## Abstract

We demonstrated an unambiguous quantum dot cascade laser based on InGaAs/GaAs/InAs/InAlAs heterostructure by making use of self-assembled quantum dots in the Stranski-Krastanow growth mode and two-step strain compensation active region design. The prototype generates stimulated emission at *λ* ~ 6.15 μm and a broad electroluminescence band with full width at half maximum over 3 μm. The characteristic temperature for the threshold current density within the temperature range of 82 to 162 K is up to 400 K. Moreover, our materials show the strong perpendicular mid-infrared response at about 1,900 cm^-1^. These results are very promising for extending the present laser concept to terahertz quantum cascade laser, which would lead to room temperature operation.

**PACS:**

42.55.Px; 78.55.Cr; 78.67.Hc

## Background

Quantum cascade lasers are semiconductor laser sources based on intersubband transitions in multiple quantum well systems [1]. Their unique operation principle and good performance have established themselves as the leading tunable coherent semiconductor source in the infrared and terahertz ranges of the electromagnetic spectrum [2-10]. Although quantum cascade lasers have experienced rapid development, several drawbacks still exist. First of all, the intersubbands transition nature leads to relatively narrow gain spectrum and, consequently, narrow spectrum tunability [11]. Moreover, due to intersubband selection rules, the emitting light is polarized in the growth direction, which makes surface emission impossible. Another drawback is that due to numerous in-plane scattering paths that the electrons undergo and decrease the upper lasing state lifetime, the threshold current is increased and the wall plug efficiency is decreased [12-17]. An appealing and ambitious route to tackle these difficulties is to explore quantum dot cascade laser (QDCL) [17,18], by substituting the quantum wells (QWs) in the active region with self-assembled quantum dots (QDs).

The development of QDCL using self-assembled QDs as substitute for QWs in the active region faces two challenges: (1) the QDs' size and controllability, implying the effective of three-dimensional (3D) quantum confinements, i.e., the prerequisite of realizing the ‘phonon bottleneck’ effect and (2) the adjustable energy levels, which satisfy critical requirements of injection and extraction efficiency. Here, our design targets precisely these challenges: first, two-step strain compensation mechanics using InGaAs/GaAs/InAs/InAlAs material system can realize controllable InAs QDs on tensile-strained InAlAs layers; second, the population inversion is achieved between lower levels of coupled InAs QDs and upper hybrid QW-dominated lasing states.

## Methods

**Figure 1:**
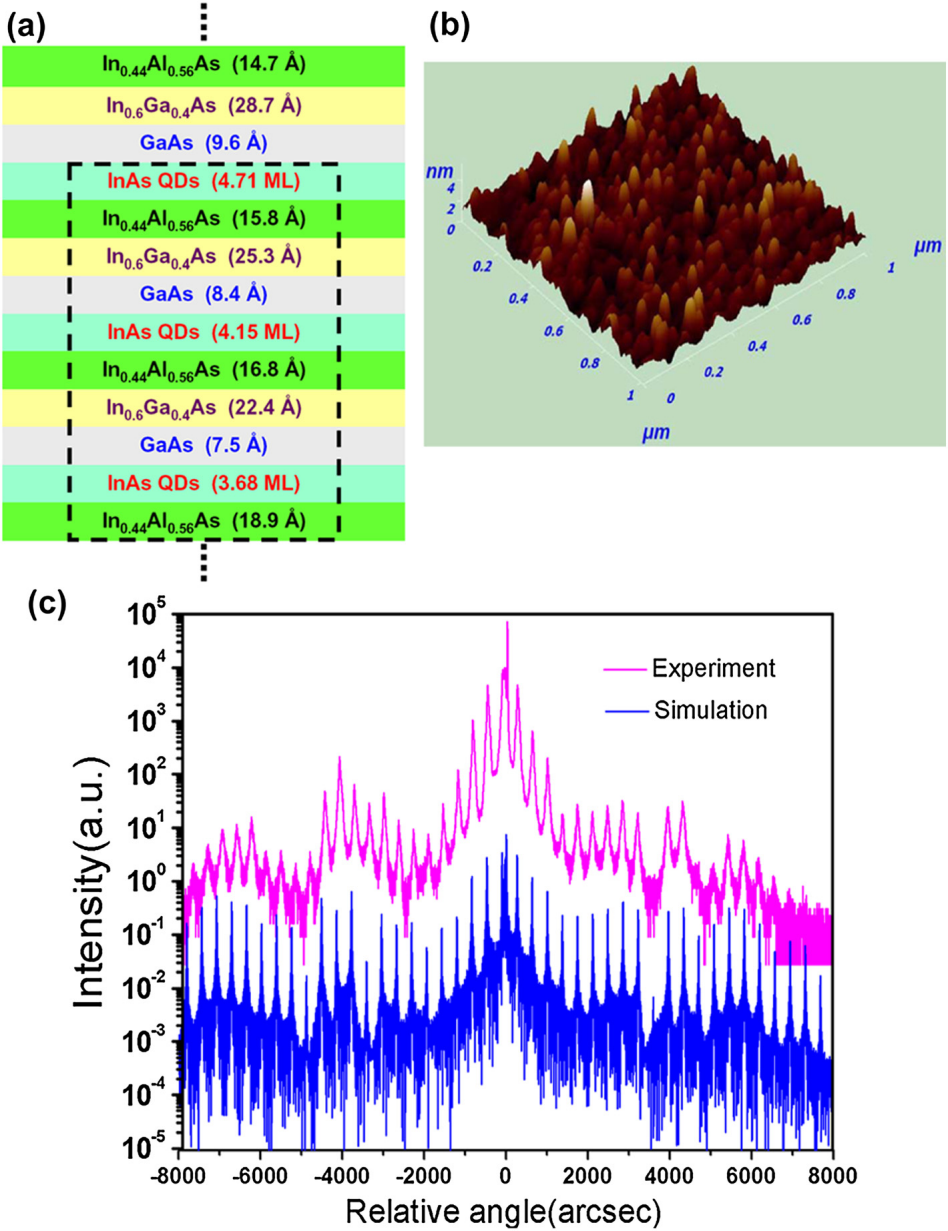
**Active region structure, AFM image, and XRD curves. (a)** Self-assembled InAs QDs grown by two-step strain compensation mechanics. **(b)** AFM image of coupled InAs QDs (dashed rectangle in Figure 1a on top of one period InGaAs/GaAs/InAs/InAlAs QDCL active region). **(c)** Experimental and simulated X-ray diffraction rocking curve for a 30-stage QDCL structure.

Considering that InAs QDs grown on GaAs/AlGaAs material system [19-21] lack of a suitable extraction mechanism from the levels confined in the QDs and InAs QDs grown on InP-based InGaAs/InAlAs material system [22-27] tend to be quantum dashes due to lower strain and the influence of embedding material, the radical way to realizing controllable InAs QDs in the active region is illustrated in Figure 1.

Figure 1 depicts the growth mechanics of coupled InAs QDs in the QDCL wafer. In order to restrain the appearance of unavoidable InAs quantum dashes on In_0.53_Ga_0.47_As, In_0.52_Al_0.48_As, and In_0.53_Al_0.24_Ga_0.23_As layers lattice-matched to InP substrate, the InAs QDs are grown on tensile-strained In_0.44_Al_0.56_As and caped by GaAs to increase the lattice mismatch between InAs and embedding materials system. This GaAs/InAs(QDs)/In_0.44_Al_0.56_As triple layer is a QDs-embedded composite layer which is partially strain-compensated, but still tensile-strained as a whole. This approach points out that the distillation of the first step of the two-step strain compensation mechanics brings on two advantages: the feasible route for forming self-assembled InAs QDs and the flexibility in quantum engineering. The second step of two-step strain compensation mechanics is using In_0.6_Ga_0.4_As layers to compensate the QDs-embedded composite layers in active region and using In_0.6_Ga_0.4_As/In_0.44_Al_0.56_As layers in the injection/collection regions, aiming at strain compensation in one period of QDCL.

The QDCL structure was grown by molecular beam epitaxy (MBE) combined with metal-organic chemical vapor deposition (MOCVD). The epitaxial layer sequence starting from the n-doped InP substrate was as follows: 1.3 μm InP cladding layer (Si, 2.2 × 10^16^ cm^-3^), 0.3-μm-thick n-In_0.53_Ga_0.47_As layer (Si, 4 × 10^16^ cm^-3^), 30 QDCL stages, 0.3-μm-thick n-In_0.53_Ga_0.47_As layer (Si, 4 × 10^16^ cm^-3^), 2.5 μm upper cladding (Si, 2.6 × 10^16^ cm^-3^), and 0.6 μm cap cladding (Si, 1 × 10^19^ cm^-3^). The active core of QDCL is based on a bound-to-continuum design. The layer sequence, with four material compositions, starting from the injection barrier is as follows (in angstroms, and InAs in monolayer (ML)): **44.1**/13.7/**14.7**/28.7/***9.6***/*4.71ML(InAs)*/**15.8**/25.3/***8.4***/*4.15ML(InAs)*//**16.8**/22.4/**7.5**/*3.68ML* with In_0.44_Al_0.56_As in bold, In_0.6_Ga_0.4_As in regular, GaAs in bold and italic, and InAs QD layer in italic style, and underlined layers correspond to the doped layers (Si, 1.5 × 10^17^ cm^-3^). Only InP was grown by MOCVD. For InAs QDs, the nominal growth rate was 0.41 ML/s, and the substrate temperature was kept at 510°C during MBE growth. After the QD layer was deposited, 30 to 60 s of ripening time was given under As_4_ protection.

The wafer was processed into double-channel ridge waveguides using conventional photolithography and wet chemical etching. The detail of fabrication is identical to [28]. The average core width is 16 μm, and the waveguides were cleaved into 3-mm-long bars. The laser spectral measurements were carried out using two Fourier transform infrared (FTIR) spectrometers (Bruker Equinox 55 Bruker Corporation, Billerica, MA, USA; and Nicolet 8700, Thermo Fisher Scientific, Hudson, NH, USA). The emitted optical power from laser was measured with a calibrated thermopile detector placed directly in front of the cryostat with a corrected collection efficiency of 15%.

In order to demonstrate the role of QDs in the active region further, we also performed the subband photocurrent measurements. The wafer was processed into circular mesa with a diameter of about 340 μm using conventional photolithography and wet chemical etching. The etch depth was down to the substrate. The Ti/Au ohmic contact was deposited onto the top surface of the circular mesa as the top contact with a diameter of 170 μm and onto the upper surface of the substrate as the bottom contact. For the PC measurements, the incident light, namely, the infrared (IR) beam from the FTIR spectrometer, was perpendicular to the mesa upper surface; and for our structure on the mesa upper surface, the area exposed to the light occupies about 75% of the total area.

## Results and discussion

**Figure 2:**
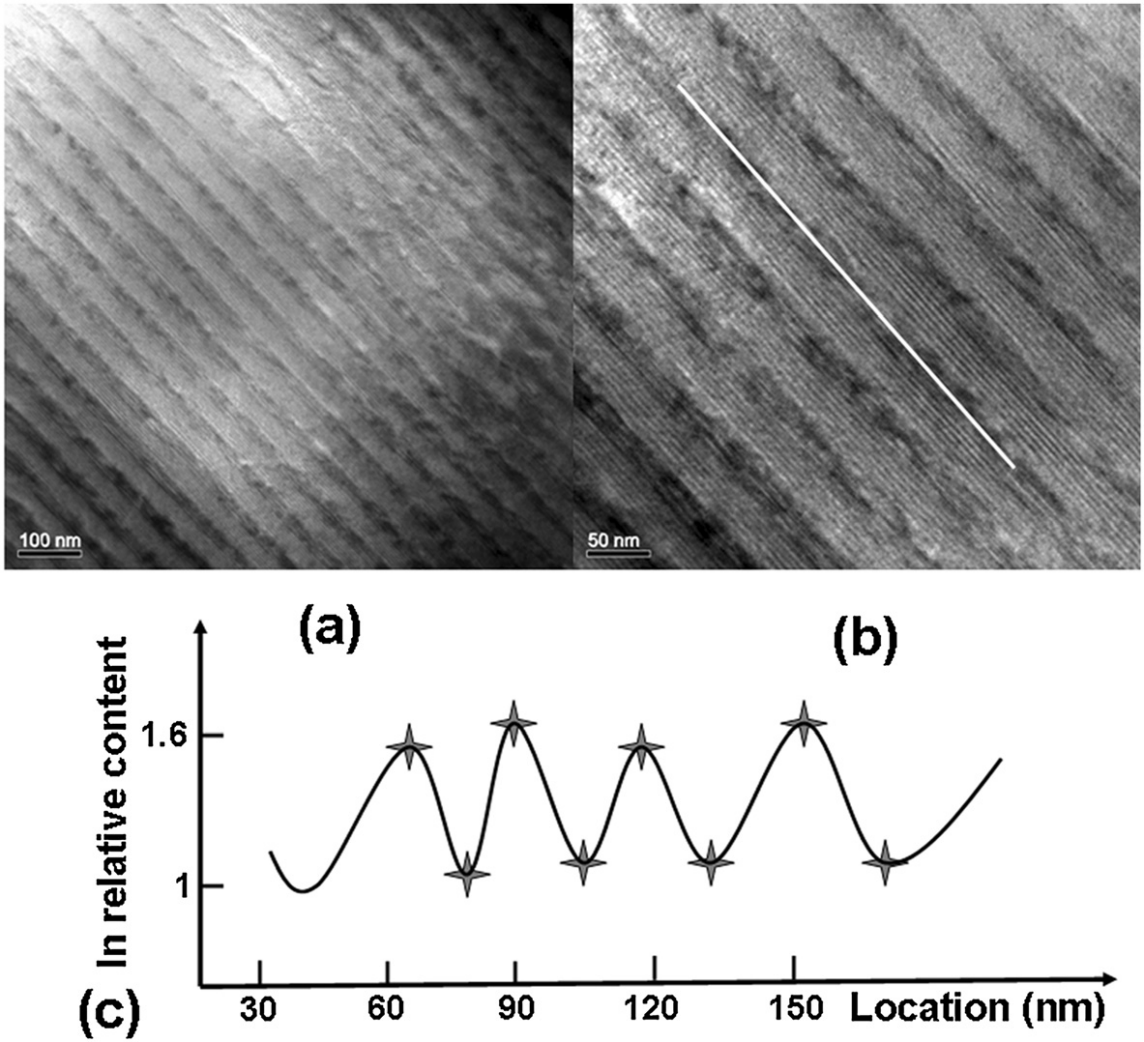
**TEM image and EDS results. (a)** TEM image of a portion of the cleaved cross section of a QDCL active region. **(b)** The enlargement image of a portion of Figure 2a for clarity, and the white line gives a clear indication of QDs distribution parallel to the growth layer. **(c)** Indium relative content along the indicated white line in Figure 2b measured by X-ray energy dispersion spectra.

Figure 1a gives the scheme of one unit of coupled QDs lasing layers in one period. Figure 1b shows the atomic force microscopy (AFM) image of one-period QDCL with another unit of coupled QDs lasing layers (indicated by the dashed rectangle in Figure 1a) on top. The average diameter of QDs is about 30 nm, with a height of 2.5 nm. The entire structural quality of the QDCL wafer was confirmed by the X-ray diffraction (XRD) spectrum as shown in Figure 1c. In the XRD simulation, we treated the QD layer as a two-dimensional InAs layer with a homogeneous thickness corresponding to the nominal deposit amount, which was strained biaxially to match the lattice constant of InP. The experimental zeroth peak shows a nearly perfect lattice match to the InP substrate, which demonstrates that the active region layers have been properly strain-balanced to give a net zero strain. The accurate match of the simulated curve and the experimental curve shows an extremely good control over the growth parameters across the entire 30-period layer sequences. The cross-sectional view of transmission electron microscopy (TEM) images of a portion of the 30-period QDCL shown in Figure 2a,b gives the direct and clear evidences of distinct coupled QDs layers in the active core. What is more, the X-ray energy dispersion spectra (EDS) result obtained along cross section line of coupled QDs layers gives indium contents at different points. The ‘star’ represents the discrete data point of X-ray energy dispersion spectrum at each position along cross section line (Figure 2b) of coupled QDs layers of the TEM sample. Based on the finite scattered experimental data points, we sketch the continuous curve of indium composition along cross section line with periodic oscillation characteristic. The periodic oscillation characteristic of indium relative contents as shown in Figure 2c gives the additional evidence of QDs in the active region. This result is consistent with the AFM one.

**Figure 3:**
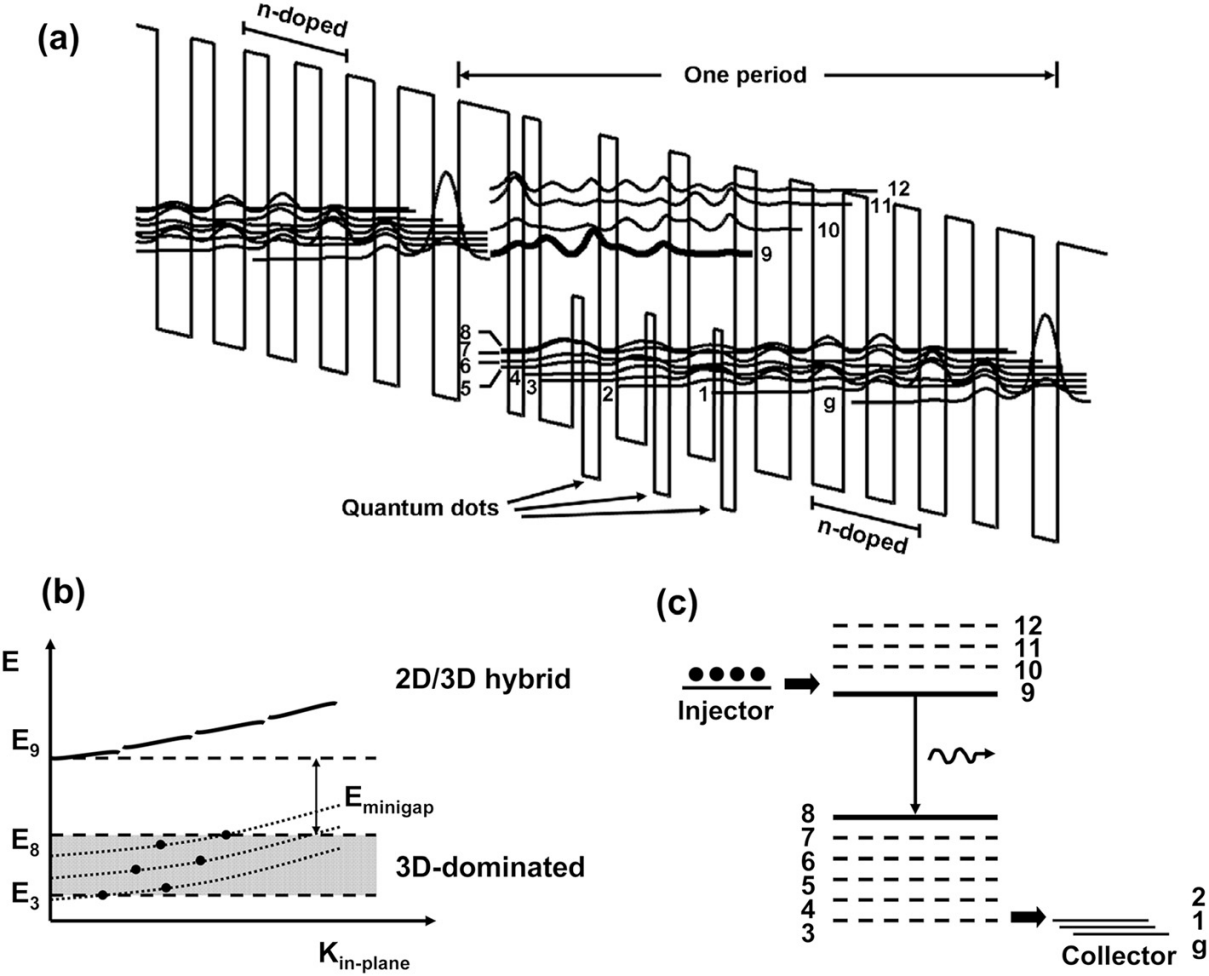
**Energy band diagram. (a)** Calculated conduction band diagrams of one period of the 30-stage QDCL active core under an electric field of 57 kV/cm using 1D model. The wavy curves represent the moduli squared of the wave functions of the relevant quantum states. The optical transition takes place between states 9 and 8. **(b)** Schematic illustration of electron energy (*E*) versus in-plane wave vector (*K*_in-plane_) relation for a period of QDCL. The in-plane state distribution is hybrid-quantized or quantized because of 3D confinement. The upper broken lines denote the hybrid-quantized states, while the lower heavy dots stand for quantized states (dotted lines indicate quasi-continuous bands of the two-dimensional confinement). **(c)** Schematic sketch of the relevant energy levels in a QDCL.

A schematic conduction band diagram of one period of the active layers is shown in Figure 3a. The design computation is based on 1D Schroedinger equation of envelope function approximation from the point of view of simplicity. The energy dependence of the effective mass and the strain effect are included in the calculation. The transition energy of 196 meV between states 9 and 8 is consistent with the experiment lasing wavelength. We also calculate the 3D coupled quantum dot states in the active region, which have about the same eigenenergy with the lower states in the simple 1D model, which implies that QD states as the final levels really contribute a lot to the electron-stimulated transition in the active region and the effectiveness of the simple 1D model.

We present here a novel design to form upper hybrid QW/QD lasing states and lower pure QD lasing states to realize the ‘phonon bottleneck’ effect. A general scheme of the electron energy versus in-plane wave vector relations is shown in Figure 3b. Although the states still have free particle-like dispersion skeleton in the direction parallel to the layers, the lateral quantum confinement breaks the subbands into quasi-continuous or discrete states. The upper hybrid subband (consists of hybrid-quantized states of QWs and QDs) is quasi-continuous, but the lower QD subband consists of widely separated in-plane energy states due to the lateral confinement of QDs. An electron in the upper quasi-continuous subband which relaxes to lower quantized states is difficult to obtain due to lack of appropriate final states. As a consequence, the relaxation time for the single-phonon process is increased. This implies that the nonradiative LO-phonon-assisted electron relaxation time in a QD is enhanced by a factor that depends on the lateral size of the QD. Figure 3c depicts the relevant energy levels and the electron injection/extraction sketch.

**Figure 4:**
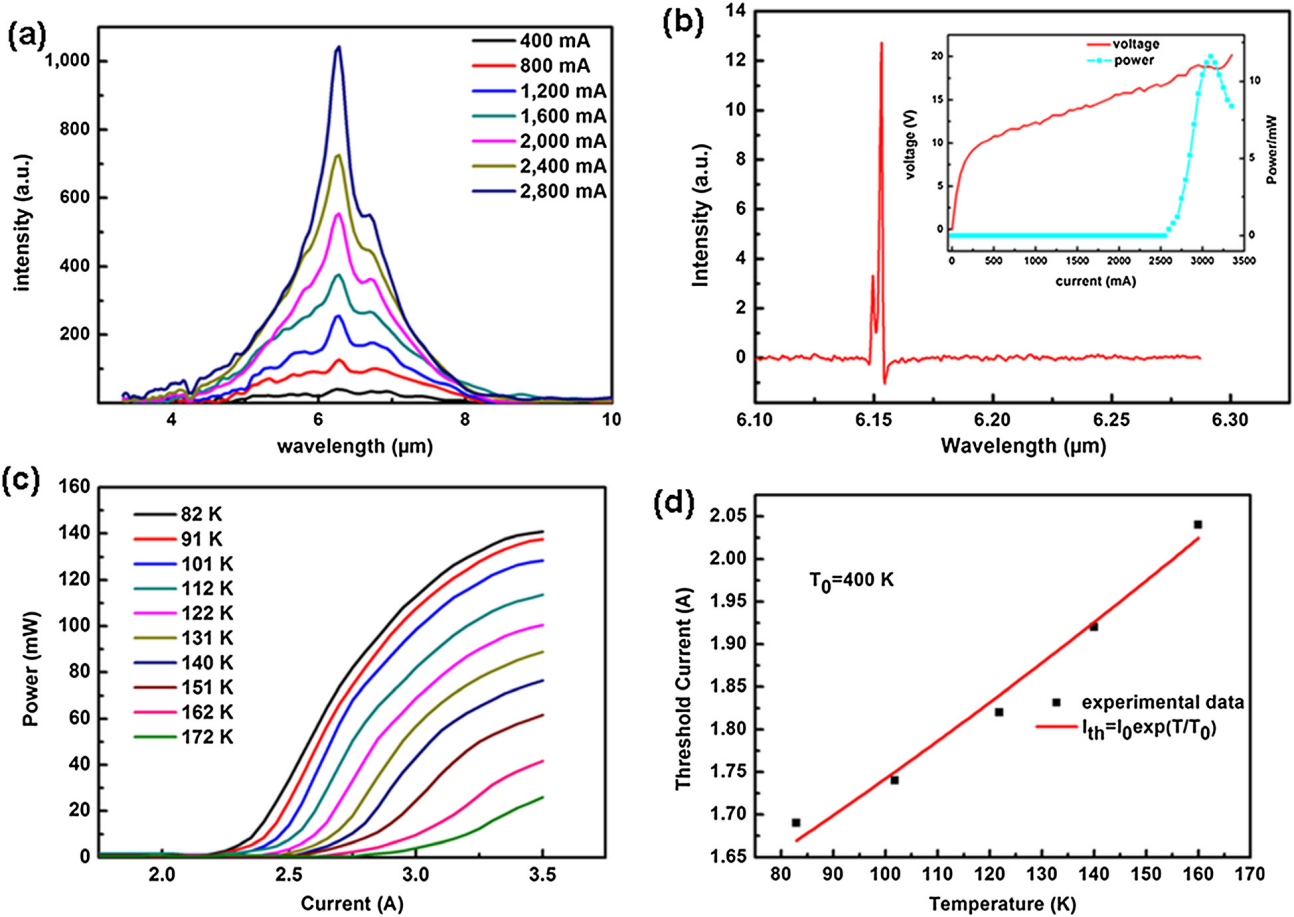
**Spectra, power, and temperature characteristics. (a)** Emission spectra from QDCL recorded at room temperature for different drive currents with a pulsed width of 1 μs and repetition frequency of 50 kHz. **(b)** Typical lasing spectrum from the QDCL recorded at 81 K with a pulsed width of 2 μs and repetition frequency of 1.5 kHz. The inset shows the voltage-current power curves. **(c)** Light-current (*L*-*I*) characteristics of QDCL operated in pulsed mode with a pulsed width of 2 μs and repetition frequency of 5 kHz. **(d)** Threshold current as a function of heat sink temperature in pulsed operation for another typical laser device. The solid curve represents fit using the empirical exponential function, *I*_th_ = *I*_0_ exp(*T* / *T*_0_).

Figure 4a shows the spontaneous emission spectra of one such laser at room temperature for different drive currents using Bruker Equinox 55 FTIR spectrometer. The spontaneous emissions at low drive currents display a full width at half maximum of 550 cm^-1^ (broad emission spectrum spanning the wavelength range of 4.5 to 7.5 μm). The very broad emission spectra confirm the typical characteristic of a broad gain medium provided by self-assembled QDs' inherent spectral inhomogeneity. We attribute the narrow peak on top of the broad base to a group of QDs in the active region with special size and then special QD energy states. These QDs are quite many in quantity, and the positions of their energy states in the energy band diagram are propitious for subsequent electron extraction after transition. Figure 4b presents typical lasing spectrum obtained at 81 K near the laser threshold utilizing Nicolet 8700 FTIR spectrometer with a resolution of 0.125 cm^-1^. Mainly stemming from the bad waveform generated by the pulsed current source (PCX-7410), we cannot get the classical multi-longitudinal-mode lasing spectra. The distinct lasing takes place at wavelength of 6.15 μm, which is consistent with the calculated transition energy of 196 meV between states 9 and 8 indicated in Figure 3a. The laser still works up to 250 K according to the spectra results of our FTIR spectrometer. However, due to the unoptimized device processing, especially the possible current leakage of SiO_2_ insulating layer under relatively high voltage (the accessorial experiment proved that the SiO_2_ layer was somewhat loose, which can lead to pinhole leakage), the prototype device cannot perform lasing over room temperature. Moreover, the voltage-current power curves as the inset of Figure 4b show the energy band alignment voltage of about 10 V.

Figure 4c shows the light power (*L*) versus current (*I*) characteristics of laser for different heat sink temperatures. A peak optical power of more than 140 mW at 82 K was measured, with a threshold current density of about 4 kAcm^-2^. The large threshold current density may stem from a number of factors, including the broad gain spectrum, the energy misalignment between injector and bound state 9, electron leakage to higher spurious states, over-discrete and inhomogeneous lower energy states due to size inhomogeneity of QDs, possible parasitical bound state between states 9 and 8, extraction efficiency of electron from low miniband not optimized, and thermal backfilling. Figure 4d shows the temperature dependence of the threshold current for another typical laser. A *T*_0_ value of 400 K is obtained within the temperature range of 82 to 162 K. This relative high *T*_0_ is also the inherent characteristic of QDs-based lasers [29-31].

**Figure 5:**
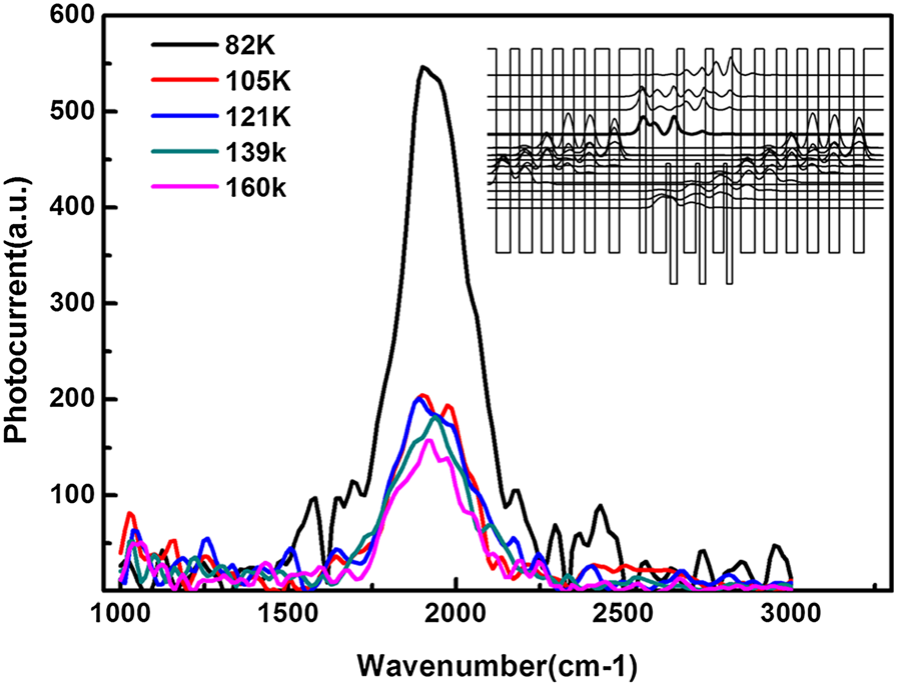
**Photocurrent spectra of samples under different temperatures and zero bias.** The PC measurements were conducted using Bruker Equinox 55 FTIR spectrometer under step-scan mode with a resolution of 16 cm^-1^. The IR beam was chopped before it arrived at the sample, and the signal from the sample was fed through a high-speed pre-amp and then input to a lock-in amplifier, which was locked into the chopper frequency. The inset shows the calculated conduction band diagram of one period of 30-stage QDCL active core under zero bias.

Figure 5 gives the PC spectra of samples under different temperatures and zero bias and demonstrates the strong perpendicular response of our materials. The spectra peak at about 1,900 cm^-1^, showing reasonable agreement with the computed result. The inset of Figure 5 is a calculated 1D conduction band diagram of one period of the 30-stage QDCL active core under zero bias from the point of view of simplicity. The energy difference between the upper lasing level (bold) and the lowest energy level corresponds to 1,790 cm^-1^. Meanwhile, we conducted some other photocurrent experiments using several normal strain-compensated quantum cascade laser (QCL) wafers with the same processing and found that their photocurrent is two or three orders of magnitude smaller than our QDCLs, which demonstrates the effect of QDs in our QDCL active region. Theoretically, normal QCL wafer does not absorb perpendicularly incident infrared light due to transition selection rule. Meanwhile, in our wafer with QDs in the active region, electrons experience the confinement from the direction in the growth plane. So according to the transition selection rule, QDCL wafer should respond to the perpendicularly incident light strongly and the experimental results confirm the QDs' effect in our sample.

## Conclusions

In conclusion, we believe that the reported structure does show quantum dot characteristics from the AFM, TEM, EDS, EL, *T*_0_, and PC measurements and to some extent, limited phonon bottleneck effects. Moreover, by improved design of the QDs-based active region of our device, in particular, aiming at the controllability on QDs size and smart two-step strain compensation, we also believe that the overall performance of QDCLs will be a great leap forward. What is more, our QDCL design concept can be transplanted to terahertz quantum cascade laser design, paving a new way for room temperature operation.

## Abbreviations

AFM: atomic force microscopy; EDS: energy dispersion spectra; FTIR: Fourier transform infrared; MBE: molecular beam epitaxy; ML: monolayer; MOCVD: metal-organic chemical vapor deposition; QDs: quantum dots; QDCL: quantum dot cascade laser; QWs: quantum wells; TEM: transmission electron microscopy; XRD: X-ray diffraction.

## Competing interests

The authors declare that they have no competing interests.

## Authors' contributions

NZ designed the laser core structure, fabricated the device, performed the testing, and wrote the paper. FQL provided the concept, grew the wafer, wrote the paper, and supervised the project. JZ, LW, and JL fabricated the device and performed the testing. SZ grew the wafer. ZW supervised the project. All authors read and approve the final manuscript.
